# Use of methane production data for genetic prediction in beef cattle: A review

**DOI:** 10.1093/tas/txae014

**Published:** 2024-01-31

**Authors:** Elizabeth A Dressler, Jennifer M Bormann, Robert L Weaber, Megan M Rolf

**Affiliations:** Kansas State University, Department of Animal Sciences and Industry, Manhattan, KS 66506, USA; Kansas State University, Department of Animal Sciences and Industry, Manhattan, KS 66506, USA; Kansas State University, Department of Animal Sciences and Industry, Manhattan, KS 66506, USA; Kansas State University, Department of Animal Sciences and Industry, Manhattan, KS 66506, USA

**Keywords:** cattle, genetic evaluation, greenhouse gas, methane, sustainability

## Abstract

Methane (CH_4_) is a greenhouse gas that is produced and emitted from ruminant animals through enteric fermentation. Methane production from cattle has an environmental impact and is an energetic inefficiency. In the beef industry, CH_4_ production from enteric fermentation impacts all three pillars of sustainability: environmental, social, and economic. A variety of factors influence the quantity of CH_4_ produced during enteric fermentation, including characteristics of the rumen and feed composition. There are several methodologies available to either quantify or estimate CH_4_ production from cattle, all with distinct advantages and disadvantages. Methodologies include respiration calorimetry, the sulfur-hexafluoride tracer technique, infrared spectroscopy, prediction models, and the GreenFeed system. Published studies assess the accuracy of the various methodologies and compare estimates from different methods. There are advantages and disadvantages of each technology as they relate to the use of these phenotypes in genetic evaluation systems. Heritability and variance components of CH_4_ production have been estimated using the different CH_4_ quantification methods. Agreement in both the amounts of CH_4_ emitted and heritability estimates of CH_4_ emissions between various measurement methodologies varies in the literature. Using greenhouse gas traits in selection indices along with relevant output traits could provide producers with a tool to make selection decisions on environmental sustainability while also considering productivity. The objective of this review was to discuss factors that influence CH_4_ production, methods to quantify CH_4_ production for genetic evaluation, and genetic parameters of CH_4_ production in beef cattle.

## Teaser

A review focused on enteric methane production encompassing the impact on the beef industry, the influence of dietary factors, quantification methodologies, and genetic parameter estimates.

## Introduction

Methane (CH_4_) is the second most abundant anthropogenic greenhouse gas (GHG) after carbon dioxide (CO_2_; U.S. EPA, 2021). In 2020, CH_4_ accounted for 11% of total U.S. greenhouse gas emissions (U.S. EPA, 2021). In the United States, 27.1% of CH_4_ emissions came from enteric fermentation by livestock species (U.S. EPA, 2021). Ruminant livestock species such as cattle, buffalo, sheep, and goats emit CH_4_ as part of their normal digestive process. Other sources of CH_4_ are landfills, animal manure, coal mining, and natural gas systems.

Greenhouse gases can be compared by their Global Warming Potential (GWP). Global Warming Potential is the amount of energy one ton of emitted gas will absorb over a specified amount of time (normally 100 yr) relative to one ton of CO_2_ (U.S. EPA, 2021). Carbon dioxide has a GWP of one while CH_4_ is estimated to have a GWP of 28 to 36. (U.S. EPA, 2021). This means that one ton of CH_4_ warms the atmosphere 28 times more than an equivalent amount of CO_2_ over 100 yr. However, GWP does not account for the differing magnitude of atmospheric burden between gases; greenhouse gases are only compared on a mass basis (Dressler et al., 2022). Methane remains in the atmosphere for far less time than CO_2_. On average, CH_4_ remains in the atmosphere for 12.4 yr, while CO_2_ remains in the atmosphere for thousands of years (Myhre et al., 2013).

Methane is composed of one carbon atom surrounded by four hydrogen atoms. The carbon atom within CH_4_ plays a role in the natural biogenic carbon cycle. The biogenic carbon cycle is centered around photosynthesis: the plant’s ability to absorb and sequester carbon. During photosynthesis, plants convert atmospheric carbon primarily into cellulose. Cattle can consume the human-inedible plant material that contains cellulose, and upcycle the carbon for growth, lactation, and other metabolic processes. As a byproduct of consuming cellulose, the carbon atom is returned to the atmosphere in the form of CH_4_ when cattle eructate. Methane remains in the atmosphere for approximately 12 yr before it is converted to CO_2_. That carbon atom is now a part of a CO_2_ molecule that plants convert to cellulose via photosynthesis and the cycle repeats again. Within the biogenic carbon cycle, the carbon atoms within CH_4_ are recycled. Therefore, no “new” carbons are released into the environment as a result of cattle producing CH_4_ (CLEAR Center, 2020). Comparatively, when fossil fuels are extracted from the Earth and burned, carbon reserved in geological storage is released into the atmosphere (Canadell et al., 2021). The accumulation of greenhouse gases in the atmosphere is dependent upon the sink–source exchange between land, ocean, and atmospheric reservoirs. Between 1750 and 2019, combustion of fossil fuels and land-use change released 700 ± 75 PgC into the atmosphere (Friedlingstein et al., 2020). However, only half remains in the atmosphere today, proving the importance of terrestrial and ocean carbon sinks in regulating atmospheric concentration (Friedlingstein et al., 2020). The objective is to review factors that impact enteric methane production, methodologies to quantify production for the purposes of genetic evaluation, and genetic parameters associated with gas production in beef cattle.

### Importance of Methane Production

The sustainability of the beef industry has been a popular topic in news and social media. Negative attention has been focused on the environmental impact of beef production. Nondairy cattle are the largest animal source of enteric CH_4_ followed by dairy cattle in the United States (FAOSTAT, 2019). Methane production by beef animals impacts all three pillars of sustainability: economic viability, environmental protection, and social equity. As a greenhouse gas, CH_4_ has an obvious impact on the environment. Methane in the atmosphere absorbs and emits radiant energy; this traps heat in the atmosphere and is why CH_4_ is considered a greenhouse gas (U.S. EPA, 2021). Social sustainability of the beef industry is entwined with the environmental impacts of CH_4_ emissions. Social sustainability includes community and organizational resilience. As a greenhouse gas, CH_4_ is related to global warming, which can disrupt the livelihoods of people by making the environment and activities within it less resilient (U.S. EPA, 2021). More broadly, CH_4_ production is tied to the impact beef production has on the viability, economy, and employment of rural communities.

Economically, CH_4_ production from enteric fermentation in beef cattle represents a decrease in efficiency for cattle production. Johnson and Ward (1996) reported that ruminants lost 5.5% to 6.5% of gross feed intake to enteric CH_4_ production. Johnson and Johnson (1995) estimate that emissions represent a loss of 2% to 12% of gross energy intake. McGinn et al. (2004) estimated that 6.5% of gross energy was lost to CH_4_ production in cattle fed a barley silage and grain diet. The amount of gross energy lost to CH_4_ production depends heavily on the acetic acid to propionic acid ratio in the rumen (Johnson and Johnson, 1995). The acetic acid to propionic acid ratio is altered primarily by feed source; fermentation of cell wall fibers, which is often observed in high-forage/roughage diets, leads to a higher acetic acid to propionic acid ratio which causes greater losses to CH_4_ production (Johnson and Johnson, 1995). Diets with a high proportion of concentrate sources, such as feedlot diets, typically lose about half the commonly cited 6% of energy to CH_4_ production (Johnson and Johnson, 1995). Thus, methanogenesis not only creates a greenhouse gas but is an energetically wasteful process. Methane produced by a ruminant does not contribute to any useful metabolic process for that animal. While CH_4_ production from ruminants will never be zero, a proportion of the 2% to 12% of gross energy lost could have rather been used by the animal for growth or maintenance.

Methanogenesis is a part of the biological process that allows ruminants to upcycle forage, therefore, maintaining animal productivity while mitigating greenhouse gas emissions is crucial. Many research studies have attempted to mitigate CH_4_ production using additives to alter rumen fermentation (and potentially animal productivity). However, it is also possible to utilize other approaches, such as genetic selection, to reduce CH_4_ emissions while also accounting for other economically relevant production traits. The advantages of this approach are that genetic improvement programs can be paired with dietary mitigation techniques, can be utilized in grazing situations where accurate administration of feed additives is substantially more difficult, and the mitigation achieved can be both permanent and cumulative (Wall et al., 2010).

According to the U.S. EPA (2021), emissions from enteric fermentation have increased by 8.4% (13.9 million metric tonnes CO_2_ Eq.) from 1990 to 2019. There were periods of time when emissions due to enteric fermentation fluctuated, but these periods usually followed the general trends in cattle population (U.S. EPA, 2021). The U.S. cattle population is smaller today compared to 1990 (USDA, 2023) therefore, total emissions per head have increased. However, Capper (2011) assessed the environmental impact of U.S. beef production in terms of resource inputs and waste outputs from 1977 to 2007. In 2007, beef production output was 82.3% CH_4_ per billion kg of beef compared to 1977 (Capper, 2011). This is largely because beef cattle efficiency increased (kg beef produced/animal; Chang et al., 2021); therefore, the total cattle population decreased. There are opportunities and challenges in the face of sustainability commitments to reduce total greenhouse gas emissions, not just emissions intensity.

### Methane Production from Enteric Fermentation

Methane is a gas that is produced through ruminant fermentation as a part of ruminant’s normal digestive processes. Microbes within the rumen work synergistically to convert forage into short-chain fatty acids and proteins (Janssen, 2010). The main products of fermentation are volatile fatty acids (VFAs) such as acetate, propionate, and butyrate (Janssen, 2010). The short-chain fatty acids are primarily absorbed across the rumen wall and provide the animal with energy which allows the animal to maintain homeostasis, reproduce, lactate, and grow. However, byproducts are produced from the fermentation process such as hydrogen, ammonia, and CO_2_ (Janssen, 2010). Methanogenic archaea in the rumen use byproducts from the fermentation process to produce CH_4_ (McGovern et al., 2020).

Hydrogen produced from the fermentation process is utilized as an energy source by methanogens to reduce CO_2_ to CH_4_ (Hunerberg et al., 2015). Another precursor to CH_4_ used by methanogens is formate, but it is used less frequently compared to H_2_ (Hungate et al., 1970). Methanogens have an important digestive function in the rumen because they are responsible for removal of H_2_, which otherwise could accumulate in the rumen and have an inhibitory effect on fermentation rate and microbial function (Van Kessel and Russell, 1996; McAllister and Newbold, 2008). After a feeding event, dissolved hydrogen increases in the rumen fluid. Smolenski and Robinson (1988) reported that the normal background hydrogen concentration of forage-fed cows was 1.0 to 1.4 µM but had peaks of 20 µM immediately following feed consumption. This is consistent with the results of Robinson et al. (1981), which reported that a cow fed grain and hay had a hydrogen concentration of 15 µM 1 h after feeding, but this value dropped to 1 µM over time. The increase in dissolved hydrogen is followed by a peak in CH_4_ production because hydrogen is a substrate for methanogenesis (Hunerberg et al., 2015).

Ruminants produce CH_4_ through fermentation in both the rumen and hindgut. According to Murray et al. (1975), 87% of CH_4_ is produced in the rumen and 13% is produced in the hindgut. Methane is released from the animal in three different ways: 1) CH_4_ produced in the rumen and hindgut is absorbed in the blood and released by expiration through the lungs, 2) CH_4_ is directly released by eructation, 3) CH_4_ is released from the hindgut in flatus (Murray et. al, 1975). Of the CH_4_ produced in the hindgut, 89% (11% of the total CH_4_ produced) is absorbed into the blood and released through expiration. Only 1% to 3% of the total CH_4_ produced is released by flatus (Murray et. al, 1975; Muñoz et al., 2012). The CH_4_ produced in the rumen is dispersed primarily by eructation and a small amount by expiration through the lungs. Most methods to quantify gas emissions exclude the small percentage of CH_4_ released in flatus and only quantify CH_4_ eructated and expired. Methodologies that do not account for CH_4_ released in flatus should still be acceptable for genetic evaluation because it is typically the animal ranking that is important rather than the raw gas production value.

Methane is emitted in pulses that vary in concentration and volume, unlike CO_2_ which is emitted more constantly (Gunter and Beck, 2018). Methane release rates for grazing ruminants follow a “diurnal biphasic pattern” with peaks in the mid-morning and late afternoon (Hegarty 2013). The peaks in emissions likely correspond with periods of more intensive grazing (Champion et al., 1994). The rate and variation of CH_4_ production throughout the day are highly influenced by feed intake. Typically, cattle that are “meal-fed” (feedlots) have CH_4_ production patterns that are different than grazing cattle because cattle that eat intermittently while grazing throughout the day have smaller changes in CH_4_ production over the day compared to “meal-fed” cattle (Gunter and Beck, 2018). Cattle that have access to ad libitum feed have more consistent CH_4_ production. Baxter and Clapperton (1965) found that sheep with constant feeding had little day-to-day variation in CH_4_ production. Accurate and high-quality data required for genetic evaluation would best be collected with a methodology that considers the diurnal patterns of CH_4_ emissions.

### Influence of Dietary Factors on Methane Production

Cattle can produce 250 to 500 L of CH_4_ per day (Johnson and Johnson, 1995). The exact amount of CH_4_ produced by an individual animal is affected by several factors, such as genetics and diet. A large variety of dietary and rumen factors have been researched and the most notable or extensively studied are discussed in the following sections.

### Rumen Conditions

Rumen pH is one factor that affects CH_4_ production. Ruminants fed diets with a high proportion of forage or fibrous plant material produce more CH_4_ per unit of feed digested (Johnson and Johnson 1995; Beauchemin and McGinn, 2005). In contrast, the addition of cereal grains/starch to a ruminant’s diet causes a decrease in CH_4_ production (Van Kessel and Russel, 1996). The addition of starch causes ruminal pH to become more acidic, which likely contributes to lower CH_4_ production because a low ruminal pH has an inhibitory effect on methanogenesis (Van Kessel and Russell, 1996). These authors found that in vitro CH_4_ production from ruminal fluid of forage-fed cows stopped when pH was <6 and hypothesized that the methanogens were not killed at an acidic pH, but entered metabolic stasis. However, Hunerberg et al. (2015) conducted an in vivo study that measured pH using indwelling pH loggers and observed that a pH as low as 5.2 did not inhibit CH_4_ production. Daily mean CH_4_ emission and ruminal pH had a low coefficient of determination (*R*^2^ = 0.27; Hunerberg et al., 2015). Because of the low *R*^2^, factors other than ruminal pH such as propionate formation and passage rate likely contributed to the lower CH_4_ emissions in cattle fed high-grain diets compared to high-forage diets (Hunerberg et al., 2015). When cattle are suddenly switched from a forage to grain diet, there is a dramatic drop in ruminal pH, which is called acute acidosis (Owens et al., 1998). During acute acidosis, the fermentation end-products are altered from acetate, propionate, and butyrate to primarily lactate, and methanogenesis is paused (Van Kessel and Russel, 1996; Hunerberg et al., 2015). However, intentionally inducing acute acidosis by altering diets to include more grains is not an efficient CH_4_ mitigation strategy due to the negative impact on animal health (Hunerberg et al., 2015).

Another factor that affects CH_4_ production is ruminal passage rate or feed disappearance rate. The ruminal passage rate is controlled by the feed type but also by the individual animal to some extent (Janssen, 2010). In general, concentrate feeds have a higher passage rate than forage feeds, meaning forages move through the rumen and are degraded more slowly. The passage rate is lower for low quality, less readily digestible feeds. When the passage rate is higher, more emphasis is put on digestion in the abomasum and lower digestive tract. Pinares-Patiño et al. (2003) found that in a group of 10 sheep, passage rate and CH_4_ production were negatively correlated. Increasing the passage rate through the rumen is associated with lower CH_4_ production per unit of feed digested in the rumen (Janssen, 2010). This can somewhat be attributed to undigested feed passing through the rumen at higher passage rates. Higher passage rates are also associated with alternative fermentation pathways that result in more propionate and less hydrogen, and therefore less CH_4_.

### Dietary Lipids

Increased dietary fat has been shown to cause a decrease in CH_4_ production and an increase in propionate formation (Johnson and Johnson, 1995; McGinn et al., 2004). The addition of long-chain polyunsaturated fatty acids to a diet decreases methanogenesis by providing an alternative hydrogen acceptor for the reduction of CO_2_ (Czerkawski et al., 1966). The addition of supplemental fat such as animal tallow or soybean oil causes decreased CH_4_ production (Van der Honing et al., 1981), though the reduction of CH_4_ was attributed to decreased fermentable substrate in this study. McGinn et al. (2004) found that when steers fed a forage-based diet were supplemented with sunflower oil, gross energy lost to CH_4_ production decreased by 21%. The addition of sunflower oil was also associated with a 20% decrease in total-tract digestibility of neutral detergent fiber (NDF; McGinn et al., 2004). The reduction in fiber digestion is likely what caused the decrease in CH_4_ production. In contrast, Hales and Cole (2017) observed that animals fed the diet with the highest dietary fat (steam-flaked corn with 45% wet distillers grains with solubles [WDGS]) in their experiment had the highest hourly and total CH_4_ production. The reason for this discrepancy could be the source of fat. The diet with the highest CH_4_ production in this study used WDGS as the primary fat source. The inclusion of WDGS in a diet increases fat concentration but also increases the proportion of protein and NDF, which is likely what caused the increase in hourly CH_4_ production for this diet (Hales and Cole, 2017). Several factors affect CH_4_ production, but the sources and interactions between these factors are important for an accurate prediction/assumption of CH_4_ production.

### Feed Type and Processing

Feed processing technique influences the passage rate and is therefore another factor that affects CH_4_ production. Blaxter (1989) found that grinding and pelleting a forage source decreased CH_4_ production because processing the forage increased the passage rate. Processing feed results in smaller particle sizes so feed exits the rumen more easily and microbial degradation is faster (Janssen, 2010). Hales and Cole (2017) tested hourly CH_4_ production rates when feeding diets composed of corn with two types of processing (dry-rolled or steam-flaked) and WDGS inclusion rate. Starch digestibility increases when corn is more processed. The inclusion of WDGS prolonged peak CH_4_ production, whereas cattle fed diets with steam-flaked corn resulted in earlier peak CH_4_ production (Hales and Cole, 2017).

More propionate is produced as a proportion of total fermentation products when a ruminant is fed a grain diet compared to a forage diet (Beauchemin and McGinn, 2005). Tajima et al. (2000) observed that rumens of cows that were consuming a grain diet contained more bacteria in the *Selenomonas–Succiniclasticum–Megasphaera* group in *Clostridium* cluster IX than cows fed forage diets. This group of bacteria produces more propionate as a major fermentation product (Janssen, 2010). Increases in propionate formation are strongly associated with decreased CH_4_ production because propionate production and methanogenesis are competing pathways (Beauchemin and McGinn, 2005; Janssen, 2010).

### Alternative Hydrogen Sinks

The reduction of CO_2_ to CH_4_ is usually the major H_2_ sink, however, other inorganic compounds such as nitrate, fumarate, and sulfates can serve as alternative H_2_ sinks (Nolan et al., 2010; Van Zijderveld et al., 2010, 2011). Nitrate has been identified as an alternative nonprotein nitrogen source to urea and has been shown to reduce enteric CH_4_ emissions in ruminants (Nolan et al., 2010). In fact, nitrite and ammonia formation are favored over CH_4_ production when nitrate is present in the rumen (Ungerfeld and Kohn, 2006). Velazco et al. (2013) supplemented feedlot steers with either urea or nitrate and measured gas fluxes with the GreenFeed system (C-Lock, Inc., Rapid City, South Dakota). Dietary nitrate supplementation significantly reduced CH_4_ yield (MY; g CH_4_/kg DMI; Velazco et al., 2013). However, there are disadvantages to feeding alternative H_2_ acceptors to lower CH_4_ production. Nitrate lowers the palatability of diets because it is bitter, which raises concerns about lowering feed intake (Lee et al., 2015). Another concern with using nitrate or sulfate in feed supplements to lower CH_4_ production is the risk of nitrite toxicity and polioencephalomalacia, which can cause death in cattle (Cammack et al., 2010; Cockburn et al., 2013).

### Ionophores

Ionophores are another dietary factor that can influence the amount of CH_4_ cattle produce (Beauchemin et al., 2008). Ionophores are a class of antibiotic-like compounds that can be added to feed with the benefits of increased feed efficiency and decreased risk for bloat, acidosis, and coccidiosis (Byers, 1980; McGuffey et al., 2001). Ionophores alter the fermentation process by decreasing the acetate:propionate ratio and the ruminal protozoa population (Ellis et al., 2012) which is why CH_4_ production is also impacted. A meta-analysis by Appuhamy et al. (2013) reported that monensin, a type of ionophore, reduced CH_4_ emissions by 19 ± 4 g/animal/d in beef steers across 22 studies. However, the use of ionophores for long-term CH_4_ mitigation is not likely because the inhibitory effects on CH_4_ emissions are usually temporary (Johnson and Johnson, 1995; Guan et al., 2006).

### Rumen Microbiome Composition

The host-rumen microbiome relationship is an important factor in enteric CH_4_ production. A subset of the rumen microbiome called the “core microbiome” is heritable and associated with CH_4_ emissions (Difford et al., 2018; Wallace et al., 2019; Martinez-Alvaro et al., 2022). Wallace et al. (2019) identified several operational taxonomic units in the core microbiome that were associated with VFA concentrations and CH_4_ production traits. Selection based on 30 of the most informative microbial genes for CH4 production led to mitigation of 7% to 17% of the mean CH_4_ emissions per generation (depending on selection intensity; Martinez-Alvaro et al., 2022). Additional research is warranted to evaluate the potential of microbiome-targeted CH_4_ mitigation and its incorporation into breeding strategies.

### Methods to Quantify Methane Production

Several strategies to mitigate CH_4_ emissions have been researched related to diet such as supplementation with fats (McGinn et al., 2004), ionophores (Appuhamy et al., 2013), direct-fed microbials (Ghorbani et al., 2002), nitrate (Nolan et al., 2010) and others. However, genetic selection is a mitigation strategy that would result in permanent and cumulative change. The pairing of genetic selection and dietary solutions could have additive mitigation effects for CH_4_ production as seen possible for other traits like feed efficiency. However, more research is needed in this area to elucidate the mitigation potential of this strategy and the interactions between all factors involved.

Large-scale research to accurately quantify enteric CH_4_ production phenotypes is crucial for genetic evaluation. There are several methods to quantify CH_4_ production from cattle including prediction models, respiration calorimetry, the sulfur-hexafluoride tracer technique, infrared spectroscopy, and the GreenFeed system. Each method has distinct advantages and disadvantages for phenotype data collection to be used in genetic evaluations.

### Prediction Models

Mathematical models can be used to predict CH_4_ emissions rather than directly measuring CH_4_ emissions from animals by a quantification technique. These models can be classified as either empirical/statistical or mechanistic (Kebreab et al., 2016). Empirical models directly relate nutrient intake to CH_4_ emissions whereas mechanistic models predict CH_4_ emissions by simulating the underlying process of fermentation (Kebreab et al., 2016).

Measuring CH_4_ production is often expensive and requires complex equipment. An advantage to using prediction models is that models do not require any additional equipment to quantify CH_4_ (Kebreab et al., 2016). In addition, empirical prediction models are relatively simple and require fewer input variables, making empirical models a more practical option compared to mechanistic models (Appuhamy et al., 2016).

Numerous prediction models exist with a range of different data inputs, from DMI to milk production characteristics. For example, Dijkstra et al. (2011) developed CH_4_ prediction equations based on milk composition for dairy cattle. Milk fatty acids are suggested to be an indicator of rumen conditions and CH_4_ production because certain fatty acids are absorbed into the blood and can be used in the mammary gland for milk fatty acid production (van Engelen et al., 2015). Other prediction models use input variables such as body weight, DMI, or feed characteristics such as total digestible nutrients (Moe and Tyrrell, 1979; Mills et al., 2003; Uemoto et al., 2020).

However, there are several limitations to prediction models. A meta-analysis of greenhouse gas emissions models reported that the predictive power of the model depends upon the accuracy of the mathematical equation and the data inputs used in that equation (Kebreab et al., 2016). Errors in estimating feed intake, stoichiometry of VFAs, and ruminal fermentation conditions were identified by Bannink et al. (2011) as the most likely sources of uncertainty in mechanistic models. Kebreab et al. (2016) advised that in a pasture system, empirical models are not good predictors of CH_4_ because DMI cannot be reliably measured in these systems. Empirical models often include a measure of feed intake which is often not available for individual animals in a commercial operation (Hristov et al., 2018). Another disadvantage of prediction models is that the model assumptions may not be met in all situations, especially if applied to a commercial livestock operation (Kebreab et al., 2016). For example, one assumption is that animals are healthy and not affected by environmental conditions. However, this ideal scenario is rarely representative of all animals.

One of the biggest drawbacks of prediction models is that prediction models do not provide individual animal information distinct from differences in feed intake (Lakamp et al., 2022). A variable that represents feed intake is always included in a prediction model. Therefore, CH_4_ production is calculated as a function of feed intake and there is no opportunity to identify and select animals which have lower CH_4_ emissions than other animals at the same feed intake amount. Additionally, it is possible that when selection is changing a population, the relationship between variables in a model could change causing the model coefficients to be less accurate at predicting gas emissions. The model would need to be re-analyzed and validated for accuracy over time.

In general, prediction models can be useful, especially if all necessary variables are readily available or quantification equipment is not available. Prediction models are probably best applied to efforts to estimate CH_4_ production from large groups of animals where mean production and feed intake are likely to be accurate for the entire group. Prediction models are less useful to predict CH_4_ from individual animals, such as would be necessary for genetic evaluation, for which a gas quantification technology would be preferred.

### Respiration Calorimetry

Respiration calorimetry techniques include whole-animal chambers, head boxes, ventilated hoods, and face masks. Respiration chambers are considered the benchmark for CH_4_ emission measurement, though every system has its strengths and weaknesses. Respiration chambers are a whole-animal open-circuit “room” used to measure respiratory exchange and gas fluxes. Inflowing air is circulated in the chamber and mixed with emitted gases. The amount of gas emitted can be found by comparing the concentration of that gas in the incoming and outgoing air (Hammond et al., 2016).

The main advantage of whole-animal respiration chambers is that it is one of the only techniques that capture both ruminal and hindgut CH_4_ emissions. Whole-animal respiration chambers capture the estimated 1% to 3% of emissions that occur in flatus (Murray et al., 1975; Muñoz et al., 2012). Respiration hoods and headboxes do not capture hindgut CH_4_ emissions. However, respiration chambers are expensive and labor-intensive (Arthur et al., 2017). They are expensive to construct and maintain, and extensive labor is required for animal training and care (Johnson and Johnson, 1995). These factors often limit the number of animals that can be measured. A sufficient sample size is imperative for genetic improvement studies, so these systems pose a major limitation to that work. Studies that use respiration chambers generally have high-quality data, provided gas recovery tests are satisfactory, but require more time and resources to obtain a sufficient sample size compared to other techniques.

Respiration chambers require the animal to be removed from their normal environment and housed individually. This often causes changes in animal behavior and reduced dry matter intake (DMI). For example, in a study done by McGinn et al. (2004), steers were moved from their normal outside pens to the respiration chambers, resulting in a decrease in DMI of 15% to 19%. Sheep in respiration chambers had 15% to 25% lower feed intake compared to their feed intake from the previous week in individual indoor home pens (Bickell et al., 2014). The decrease in DMI associated with respiration chambers is likely due to the stress of handling and their new environment. Animals using a respiration chamber can experience stress from relocation and feeding pattern disruption. A lower DMI leads to an underestimation of CH_4_ emission, which may be the most severe in the most stressed animals, confounding two different traits. Therefore, the CH_4_ production observed in a respiration chamber can be lower than the actual production in the animal’s normal environment, resulting in an underestimation of CH_4_ production of individuals, and more broadly if used in a life cycle assessment, for example.

Respiration chambers have two main sources of variation: airflow rate and air mixing in the chamber. Gardiner et al. (2015) reported that ducting/airflow and chamber mixing have 15.3% and 3.4% variability between the results of different facilities, respectively. For respiration chambers to be accurate, they must be calibrated and have a 100% gas recovery rate (Gardiner et al., 2015). Thus, respiration chambers must be airtight sealed to prevent undesigned air leakage. Modern respiration chambers are designed so that the door must be open for up to 1 h every day for animal feeding, milking, cleaning, etc. (Hristov et al., 2018). Depending upon the design of the entire respiration chamber system, air sampling may occur even more infrequently. Typically, the time “gap” is excluded from data analysis. However, this practice could lead to inaccurate CH_4_ measurements because CH_4_ emission is not constant throughout the day. Methane emissions follow a diurnal pattern dependent upon time of feed intake and have significant hour-to-hour variation. For dairy cows, the peak hourly rate of emission can be three times greater than the minimum hourly rate (Hristov et al. 2018). Whole-animal respiration chambers do not necessarily have continuous measurement and excluding periods of time from data analysis, especially near feeding time, could lead to bias in measurements.

The environment in the respiration chamber is artificial and does not represent a production environment. Energy expenditure from animals in respiration chambers is lower than in a typical production setting due to a reduction in movement space and less exposure to variable environmental conditions (Arthur et al., 2018). A reduction in energy expenditure is another possible reason that DMI is reduced for animals in respiration chambers (Llonch et al., 2016). Respiration chambers, while generally being highly accurate, lack application in a production environment and have several characteristics that inherently make phenotype collection for genetic evaluation difficult.

### Sulfur-Hexafluoride Tracer Technique

The sulfur-hexafluoride (SF_6_) tracer technique was one of the first techniques developed to measure gas emissions in an open-air environment without confinement. The SF_6_ technique was developed in the early 1990s by Patrick Zimmerman (Zimmerman, 1993). Sulfur-hexafluoride is used as a tracer gas to measure CH_4_ emissions of ruminants because it is synthetic and not produced in any biological process. It is also easily measured and traceable at low concentrations, a requirement for a tracer gas technique (Hill et al., 2016). An inert bolus containing liquid SF_6_ is placed in the rumen of the animal. The SF_6_ is slowly released from the bolus in gaseous form through permeations in the bolus at a known rate. The animal wears a halter with a capillary tube that is connected to an evacuated sample container on its back, or an inflatable neck collar. The vacuum in the sampling container collects the metabolic and tracer gas from the nose and mouth. After the trial, CH_4_ and SF_6_ concentrations are determined using the known permeation rate of SF_6_ from the bolus and the mixing ratio of gases collected in the sampling container (Zimmerman, 1993).

The advantage of the SF_6_ technique is that animals are not required to be restrained or enclosed in a chamber (Gunter and Beck, 2018). Therefore, animals are free to move and graze in their normal environment. There are several disadvantages of using the SF_6_ technique. One disadvantage is the extensive labor required. The animals must be trained to wear the halter and the sampling container, which is laborious (Gunter and Beck, 2018). In addition, labor is required to insert the bolus into the animal’s rumen. Due to these reasons, this technique is typically only used in short-duration trials with a small number of animals, which limits possible applications for genetic improvement. Another disadvantage is that this technique does not account for CH_4_ released as rectal flatus (Murray et al., 1975; Gunter and Beck, 2018). The SF_6_ tracer technique can bias the CH_4_ measurements due to the diurnal patterns of CH_4_ emissions (Gunter and Beck, 2018). The collection canisters are removed and replaced every 24 h. The rate of sample collection is not constant and sample volume is greater when the canister is first activated than it is after 24 h (Berndt et al., 2014). If the canisters are replaced during a distinct bout of feeding and subsequent increased CH_4_ emission, the greatest sampling rate occurs when CH_4_ emissions are the greatest, leading to an overestimation of emissions (Berndt et al., 2014).

The SF_6_ tracer technique is dependent upon low background gas concentrations so that differences in CH_4_ emissions can be detected (Williams et al., 2011). Typically, additional canisters called local background gas canisters are placed around the research area to correct for background gas concentration. However, if this technique is used on animals housed indoors, large variability in background gas concentration is often an issue due to poor ventilation (Williams et al., 2011; Dorich et al., 2015). Variability in background gas concentration negatively impacts the precision of the SF_6_ tracer technique.

### Infrared Spectroscopy

Infrared spectroscopy is a method to measure CH_4_ that is primarily used in dairy cattle. One method, Fourier transform infrared (FTIR), uses the infrared transmission spectrum to identify an absorbance spectrum from an air sample (Teye et al., 2009). Then CH_4_ densities can be calculated for each sample using the absorbance spectrum. Another infrared spectroscopy method of CH_4_ concentration quantification is based on mid-infrared spectra. Infrared spectroscopy methods have the advantage that they are noninvasive, and animals can remain in normal production environments during collection. Lassen and Løvendahl (2016) used the mean CH_4_:CO_2_ ratio from an FTIR unit and calculated CO_2_ emissions based on milk yield, liveweight, and days carried calf (Madsen et al., 2010) to estimate daily CH_4_ emissions. Measurements from infrared spectroscopy are highly variable, and several hundred measurements are required during a short period of time to quantify the mean of the dependent variable for individual animals (Lassen and Løvendahl, 2016). However, daily CH_4_ output measured in respiration chambers and estimated using infrared spectroscopy had a linear relationship (*R*^2^ = 0.79) for a group of 12 Holstein cows (Garnsworthy et al., 2012). The method is primarily used for dairy cows because the data can be collected during times of feeding or milking.

### The GreenFeed System (C-Lock Inc.)

A GreenFeed is an automated and voluntary head box system that quantifies gas fluxes by exhausting air past the animal’s head and into the system (Hristov et al., 2015). The GreenFeed system (C-Lock, Inc.) is currently the only product of this kind on the market for commercial use. The GreenFeed measures individual CH_4_, CO_2_, hydrogen, and oxygen gas fluxes for a variety of livestock species. The GreenFeed entices animals to visit the unit multiple times a day by releasing a small amount of pelleted feed as bait. Individual animals will insert their head into the hood and the unit will measure these gas fluxes as air is continuously drawn past the head of the animal. Eructated gas concentrations are compared to the background gas concentration. The GreenFeed collects several short-term breath samples throughout the day to calculate gas production rates (Herd et al., 2020). Measurements are an accumulation of spot samples, unlike the continuous sampling of respiration chambers and the SF_6_ technique.

One of the main advantages of the GreenFeed is that data can be collected on grazing animals in a pasture setting. It is ideal for CH_4_ emissions of grazing animals to be determined at grazing so that data can be representative of a forage diet and grazing behavior (Waghorn et. al., 2016). In contrast, cows in respiration chambers fed cut pasture have different forage and feeding patterns compared to grazing animals (Waghorn et al., 2016) which will affect methanogenesis (Jonker et al., 2014). Another advantage of the GreenFeed is that animals are unencumbered by respiration equipment or respiration chambers and do not require extensive training.

A disadvantage of the GreenFeed system is that spot samples throughout the day are combined to calculate the daily CH_4_ production, as detailed in Huhtanen et al. (2015). In other words, CH_4_ production is not continuously measured during the whole day as it would be for the respiration chamber or the SF_6_ tracer technique. One concern with the GreenFeed is if the spot samples throughout the day can capture the variation in CH_4_ emissions due to circadian rhythms. Hammond et al. (2016) recommended that a sufficient number of samples and sampling times be included in the sampling protocol to account for diurnal variation of emissions. However, Ryan et al. (2022) observed a difference of 98 g/d in mean CH_4_ between the lowest and highest emission periods of the day for cattle fed in confinement. Due to the large variation in CH_4_ production throughout the day, the authors suggested the inclusion of a time-of-day covariate in future analyses, specifically genetic evaluations.

The precision of gas flux data collected by the GreenFeed is influenced by several factors. One factor that can have a major impact on the accuracy and quality of emission estimates is the mass airflow rate. The proper airflow rate ensures that the complete breath cloud emitted from the animal is captured by the GreenFeed. Gunter et al. (2017) analyzed emission estimates with a range of airflow rates and found that when the airflow rate was above 26.0 L/s, CO_2_ and CH_4_ estimates are not affected. However, when airflow rates are below 26.0 L/s, the CO_2_ and CH_4_ emission estimates decrease (Gunter et al., 2017). These authors speculated that the reason for a decline in CO_2_ and CH_4_ emissions during lower airflow rates was due to the animals’ emitted breath cloud not being completely captured by the GreenFeed. Maintaining clean air filters for proper airflow rates is imperative for the accurate estimation of emissions.

Another important aspect of the GreenFeed that needs to be considered is feed bait delivery interval and amount of feed released. Once the animal inserts its head into the intake manifold, feed is released several times as long as the animal maintains an adequate head position. The amount of time between feed drops is the bait delivery interval and can be adjusted depending on the trial. Gunter and Bradford (2017) conducted two experiments where alfalfa pellets were fed at eight different timed intervals up to eight times per visit. Carbon dioxide and oxygen (Exp. 2 only) emission estimates were not affected by timed intervals. However, CH_4_ estimates and the ratio of CH_4_:CO_2_ linearly decreased with an increased time interval in Exp. 1, but were not different in 2 (Gunter and Bradford, 2017). In this study, increasing the time interval increased the total amount of time that animals spent in the intake manifold. Total visit duration can be increased by increasing the time interval between drops. Visits that are longer in duration more accurately capture the CH_4_ emissions from eructation events (Arthur et al., 2017).

The total amount of time that an animal spends with its head in the intake manifold is a crucial aspect of collecting high-quality data. The GreenFeed pellet delivery settings allow for visits 2 to 7 min in duration and 2 min is the minimum visit duration for a visit to be “validated” by C-Lock, Inc. Arthur et al. (2017) compared 2- and 3-min minimum visit durations. Emission records with a minimum visit duration of 2 min were significantly more heterogeneous than records with a 3-min minimum visit duration (Arthur et al., 2017). The authors concluded that to calculate an animals’ methane production rate (MPR; g CH_4_/d) and CO_2_ production rate (CPR; g CO_2_/d), a minimum visit duration of 3 min was recommended. Suggested ways to increase visit durations are 1) adjusting the interval and amount of feed drops, 2) additional training for cattle using the GreenFeed, 3) decreasing the number of animals using one unit, and 4) switching to a more palatable feed. A meta-analysis of 30 studies that used a GreenFeed reported that the actual average visit duration of animals using the GreenFeed (reported in 30% of the studies) was 3.4 min/visit and ranged from 2 to 6.6 min/visit (Della Rosa et al., 2021).

The total number of valid collection visits for an individual animal during the trial period needs to be carefully considered. Arthur et al. (2017) reported a sharp reduction in variance as more records for each individual animal were added to the dataset. The reduction in variance was so rapid that the initial variance at 5 records was reduced by over 50% after 20 records. However, after 30 records (3-min visit duration) were included in the dataset for each animal, there was no substantial reduction in variance. To achieve the same reduction in variance when the visit was only 2 min in duration, the minimum number of records needed was 40 to 45 (Arthur et al., 2017). These authors concluded that at least 30 records on an individual animal were needed to reduce variance and increase precision. Dressler et al. (2023) reported the number of visits (minimum of 2 min/visit duration) needed to accurately quantify CH_4_, CO_2_, O_2_, and metabolic rate using gases collected with a GreenFeed. The number of visits needed for CH_4_, CO_2_, O_2_, and metabolic rate were 38, 40, 40, and 36, respectively (Dressler et al., 2023). The grazing beef cows in this study took 29.5 ± 8.7 to 31.8 ± 9.2 d on average to meet the minimum number of visits recommended, depending on the gas.

There is a range of test durations recommended in the literature. Renand and Maupetit (2016) recommended a 14-d test duration with at least 50 visits to quantify CH_4_. Arbre et al. (2016) reported that a 17-d test duration would be sufficient to quantify CH_4_. Gunter and Beck (2018) reported that CH_4_ emissions could be quantified in a 14-d test period with animals visiting 2.5 times per day. The number of test days varies due to how frequently the animals visit the GreenFeed in that study. This discrepancy in the number of visits an animal makes in 1 d is one reason that spot sample recommendations should be a total number of visits for the test period instead of a test duration (Dressler et al., 2023).

Potentially due to the vast number of protocol parameters and varying protocol designs between studies, there has been inconsistency in the literature about within-animal CH_4_ repeatability using a GreenFeed. Growing Charolais bulls maintained individual ranking over four measurement periods of either 16 or 22 d each for CH_4_ (g/d), CO_2_ (g/d), H_2_ (g/d), and O_2_ (g/d; Bes et al., 2022). In this study, CH_4_ production had high repeatability coefficients of 0.61 and 0.72 (depending on diet), suggesting a consistent pattern of inter-animal variability over time. Ryan et al. (2022) measured CH_4_ production on 1,099 steers, heifers, and young bulls. Methane production was averaged across varying period lengths (within-day, 1-, 2-, 4-, 5-, 10-, and 15-d). The phenotypic repeatability of CH_4_ production ranged from 0.14 to 0.74, depending on sex and length of averaging period (Ryan et al. 2022). Although repeatability was low for averaging periods with fewer days, a repeatability of greater than 0.6 was achieved with a 10-d averaging period for steers and heifers (Ryan et al., 2022). In a study by Beauchemin et al. (2022), individual animal re-ranking was minor to moderate for CH_4_ yield (g/ kg DMI) between three 2-wk periods for crossbred beef heifers despite relatively high repeatabilities (0.56 to 0.57) between the three 2-wk periods. However, there was no re-ranking of animals with extreme CH_4_ emissions values in Beauchemin et al. (2022). This is unlike a study by Coppa et al. (2021) where individual dairy cow ranking was not stable over an 8-wk period for animals with extreme values for any of the CH_4_ production traits investigated. Further research is needed in this area to elucidate the impact of protocol design on repeatability and to weigh the cost of additional repeated measurements against the accuracy gained in genetic prediction.

### Phenotypic Methane Traits

After CH_4_ is quantified by one of the previous methodologies, various phenotypic traits for CH_4_ can be calculated (Table 1). While some CH_4_ traits vary slightly in definition, they are predominantly standardized. Individual animal CH_4_ production expressed daily is referred to as CH_4_ production or MPR which is simply the amount of CH_4_ produced by an animal per day. To account for animal productivity, other phenotypic ratio traits have been developed. Methane yield (MY) is the ratio of CH_4_ (g) over a unit of feed intake, usually DMI (kg). Methane intensity (MI) is the ratio of CH_4_ (g) over a unit of animal product. In beef cattle, MI is typically expressed over a weight measurement while in dairy cattle it typically includes a milk production trait. Additionally, several residual CH_4_ phenotypes have been developed. Residual methane production (RMP) is the actual MPR minus the expected MPR. The difficulty with RMP is calculating the expected MPR, which can be done using a regression of MPR on DMI with cohort fit as a class effect (Donoghue et al., 2016; Hayes et al., 2016). The expected MPR can also be calculated using published regression equations that usually include DMI, body weight, or milk production (Blaxter and Clapperton, 1965; Kennedy et al., 1993; Johnson et al., 1995; IPCC, 2006; de Haas et al., 2011; Dijkstra et al., 2011).

**Table 1.** Definitions of phenotypic methane traits and quantification methodologies abbreviations

	Abbreviation	Definition
Phenotypic methane trait		
Methane production rate	MPR	CH_4_ (g)/d
Methane yield	MY	CH_4_ (g)/unit of feed intake
Methane intensity	MI	CH_4_ (g)/unit of animal product
Residual methane production	RMP	Actual CH_4_ (g) – expected CH_4_ (g)
Predicted methane emissions	PME	CH_4_ (g)/d predicted with mathematical models
Quantification methodologies		
Prediction models		Mathematical models to predict emissions using component traits rather than directly measuring emissions
Respiration calorimetry		Whole-animal open-circuit systems to measure respiratory exchange and gas fluxes
Sulfur-hexaflouride tracer technique	SF_6_ tracer technique	The tracer gas (sulfur-hexafluoride) is released at a known concentration from a bolus within the rumen
Fourier transform infrared	FTIR	A type of infrared spectroscopy that uses infrared transmission spectrum to identify an absorbance spectrum from an air sample
The GreenFeed system, C-Lock, Inc.		An automated and voluntary head box system

### Comparisons Between Gas Quantification Technologies

Several studies have compared the results from the various quantification methods to each other. The enteric CH_4_ emissions of 16 Holstein cows on a common diet using the GreenFeed and the SF_6_ tracer method were compared. The GreenFeed system had relatively low coefficients of variation (14.1% to 22.4%) for CH_4_ emissions (Dorich et al., 2015). In comparison, the coefficients of variation for CH_4_ emissions using an SF_6_ were up to 5-fold greater (16.0% to 111%) than the GreenFeed (Dorich et al., 2015).

Herd et al. (2016) evaluated CH_4_ emission traits in animals with a GreenFeed and in respiration chambers. The GreenFeed was set up in feedlot conditions where animals had access to an ad libitum grain-based diet, whereas within the respiration chambers the animals had different diets in two trials: a restricted grain-based diet and a restricted roughage diet. Residual CH_4_ production and MY had moderate positive phenotypic correlations (0.54 to 0.58) between the GreenFeed feedlot test and the roughage respiration chamber test (Herd et al., 2016). In this study, the GreenFeed feedlot test was performed 73 d after the chamber roughage test, suggesting strong repeatability.

Hammond et al. (2013) compared CH_4_ production in growing dairy heifers using a GreenFeed to CH_4_ emission values measured with a respiration chamber and the SF_6_ technique. Hammond et al. (2013) reported that the GreenFeed and the respiration chambers had similar CH_4_ emission (g/d) estimates, but the SF_6_ technique estimated higher CH_4_ emission (g/d) values than the GreenFeed. The authors speculated that the higher CH_4_ emission values could have been due to different housing conditions (grazing vs. indoor) or the accuracy of the SF_6_ technique data. The patterns of CH_4_ emissions were comparable for the GreenFeed and the respiration chamber (Hammond et al., 2013).

In 2015, Hammond et al. found contrasting results to their 2013 study previously mentioned. They aimed to compare measurements from a GreenFeed to a respiration chamber and SF_6_ tracer technique. There were three experiments, all with different diets. The first two experiments used 4 animals and the third experiment used 12 animals. The GreenFeed and respiration chambers were compared in two experiments. The average CH_4_ emissions measured using the GreenFeed (198 and 208 g/d) were numerically similar to the averages from the respiration chamber (218 and 209 g/d) in the two experiments. However, the Lin’s concordance correlation coefficient (CCC) between the GreenFeed and respiration chamber CH_4_ emission (g/d) values was poor (0.1043; Hammond et al., 2015). The CH_4_ emissions from the SF_6_ tracer technique were higher than the CH_4_ emissions from the GreenFeed, but the CCC between the two was moderate (0.6017; Hammond et al. 2015). The GreenFeed did not identify the significant treatment effects on CH_4_ emissions that the respiration chamber and SF_6_ technique identified (Hammond et al., 2015). The authors suggested that the reason the GreenFeed did not detect the treatment effects was due to a limited number of animals and the timing of measurements taken with the GreenFeed.

Jonker et al. (2016) compared CH_4_ and CO_2_ measurements from a respiration chamber, a GreenFeed, and the SF_6_ technique with a small sample size of eight beef heifers. The MY estimates from the GreenFeed were similar to the estimates from the respiration chamber (Jonker et al., 2016). However, the MY estimates from the GreenFeed were greater than the estimates from the SF_6_ technique (Jonker et al., 2016). Measurement method did not affect CO_2_ values except in one period of the five periods total, in which the respiration chambers had higher values than the GreenFeed and SF_6_ technique (Jonker et al., 2016). The authors concluded that the GreenFeed system provided CH_4_ yields that were similar to the respiration chamber.

Alemu et al. (2017) grouped animals into a high-residual feed intake (RFI) or low-RFI groups based on data from the GrowSafe system (Calgary Alberta, Canada). Animals had gas flux measurements taken using both the GreenFeed and a respiration chamber. The respiration chamber had greater variation (CV = 19.9%) in CH_4_ estimates among animals compared to the GreenFeed (CV = 14.3%). Estimates for MPR for both groups and MY for the high-RFI group were greater for the GreenFeed than the respiration chamber (Alemu et al., 2017). In other words, the GreenFeed and respiration chambers had similar estimates for CH_4_ yield but different daily CH_4_ emissions (Alemu et al., 2017). The authors hypothesized the reason the GreenFeed estimated greater CH_4_ emissions than the respiration chamber was because animals in the respiration chambers had 19% to 20% lower DMI than the animals housed in group pens. The differences in CH_4_ emissions between the respiration chamber and the GreenFeed were likely due to the conditional differences between the systems, proving the difficulty in directly comparing systems without simultaneous measurement.

McGinn et al. (2021) used a novel method to compare the GreenFeed to a respiration chamber with simultaneous measurement of CH_4_ and CO_2_. Instead of using animals, a mass flow controller released known concentrations of CH_4_. The mass flow controller released CH_4_ into the GreenFeed in an open environment meant to represent a pasture setting with low background concentrations. The respiration chamber and the GreenFeed were directly compared by placing the GreenFeed inside the respiration chamber. The mass flow controller released a known amount of CH_4_ inside the respiration chamber and collected CH_4_ emission rates (g/d) from the GreenFeed and the respiration chamber. This was designed to represent a barn with the potential to have higher background concentrations. There was a significant difference between the CO_2_ emission rates between the GreenFeed and the mass flow controller (outside of the respiration chamber) and between the GreenFeed and the respiration chamber (McGinn et al., 2021). When CH_4_ emission rates were compared, there was not a significant difference between the GreenFeed and the mass flow controller. There was a small, but significant difference in CH_4_ emission rates between the GreenFeed and the respiration chamber (328 g/d vs. 323 g/d). The authors later discovered that the difference in CO_2_ emission rates between the GreenFeed and the mass flow controller was due to a systematic error. The authors concluded that the GreenFeed has the potential to accurately measure emission rates in both an open environment and a barn (McGinn et al., 2021).

Estimates quantified by the GreenFeed have also been compared to estimates calculated from prediction equations. Waghorn et al. (2013) measured CH_4_ emissions of grazing dairy cows using a GreenFeed. Methane emissions were calculated on individual animals from predictive equations based on milk production and body weight change. Measurements from the GreenFeed and calculated CH_4_ emissions had a linear relationship (*R*^2^ = 0.72).

A meta-analysis done by Huhtanen et al. (2019) aimed to compare CH_4_ production measured from the GreenFeed to CH_4_ production prediction equations. Eighteen different empirical equations based on intake and nutrient composition were selected from the literature to represent different datasets. Equations to predict CH_4_ production were selected based on the variables available in the dataset: feed intake only, intake and nutrient composition, and CH_4_ yield and feed intake (Huhtanen et al., 2019). Some datasets also included respiration chamber values which allowed for a direct comparison between the respiration chamber and the GreenFeed. The meta-analysis included 83 treatment means from both dairy and growing beef animals with a wide range of diet, nutrient composition, housing type, and environmental conditions. Huhtanen et al. (2019) found that CH_4_ production measured by the GreenFeed and predicted by the equations were closely related (*R*^2^ values in most cases > 0.90). In direct comparisons, CH_4_ production measured by the GreenFeed and respiration chamber were closely associated (*R*^2^ = 0.92) and had a high concordance correlation coefficient (CCC = 0.95; Huhtanen et al., 2019). These results suggest that there was an agreement between CH_4_ emissions measured by the GreenFeed and measurements from respiration chambers and emissions predicted by empirical models.

### Genetic Parameters

All CH_4_ quantification methodologies discussed could be used to collect phenotypes for genetic evaluation. While some methodologies have distinct advantages, they all have challenges in phenotype collection for the purposes of genetic evaluation. Collection of a sufficient number of phenotypes for genetic evaluation is expensive, time-consuming, laborious, and requires proper contemporary grouping. Therefore, estimates of heritability (Table 2) and genetic correlations (Table 3) for CH_4_ production in the literature are sparse for beef cattle.

**Table 2.** Heritability estimates of methane production traits in beef and dairy cattle

Trait	Heritability ± SE	Citation
Methane production, g/d	0.28 ± 0.06	Hayes et al. (2016) ^a^
Methane yield, g/kg DMI	0.20 ± 0.05	Hayes et al. (2016)
Residual methane production_B_^1^	0.19 ± 0.06	Hayes et al. (2016)
Residual methane production_J_^2^	0.19 ± 0.05	Hayes et al. (2016)
Residual methane production_I_^3^	0.19 ± 0.05	Hayes et al. (2016)
Residual methane production_R_^4^	0.19 ± 0.05	Hayes et al. (2016)
Methane production, g/d	0.30 ± 0.06	Manzanilla-Pech et al. (2016) ^a^
Methane yield, g/kg DMI	0.20 ± 0.05	Manzanilla-Pech et al. (2016)
Methane intensity, g/ kg weight	0.25 ± 0.06	Manzanilla-Pech et al. (2016)
Residual phenotypic methane^5^	0.19 ± 0.05	Manzanilla-Pech et al. (2016)
Residual genetic methane^6^	0.15 ± 0.05	Manzanilla-Pech et al. (2016)
Methane production, g/d	0.27 ± 0.07	Donoghue et al. (2016) ^a^
Methane yield, g/kg DMI	0.22 ± 0.06	Donoghue et al. (2016)
Residual methane production_B_^1^	0.19 ± 0.06	Donoghue et al. (2016)
Residual methane production_J_^2^	0.19 ± 0.06	Donoghue et al. (2016)
Residual methane production_I_^3^	0.19 ± 0.06	Donoghue et al. (2016)
Residual methane production_R_^4^	0.19 ± 0.05	Donoghue et al. (2016)
Methane production, g/d	0.21 ± 0.06	Lassen and Løvendahl (2016) ^b^
Methane intensity, g/L milk	0.21 ± 0.06	Lassen and Løvendahl (2016)
CH_4_:CO_2_ ratio	0.16 ± 0.04	Lassen and Løvendahl (2016)
Predicted methane yield, g/kg DMI^7^	0.12 ± 0.06	van Engelen et al. (2015) ^b^
Predicted methane yield, g/kg DMI^8^	0.20 ± 0.07	van Engelen et al. (2015)
Predicted methane yield, g/kg DMI^9^	0.44 ± 0.10	van Engelen et al. (2015)
Predicted methane emissions, g/d^10^	0.13 ± 0.04	Pickering et al. (2015) ^c^
Predicted methane emissions, g/d	0.35 ± 0.12	de Haas et al. (2011) ^b^
Predicted methane emissions, g/d^11^	0.25 ± 0.01	Kandel et al. (2017) ^b^
Daily methane production, g/d	0.19 ± 0.02	van Breukelen et al. (2023) ^c^
Daily methane concentration, ppm	0.18 ± 0.01	van Breukelen et al. (2023) ^c^
Weekly mean methane production, g/d	0.33 ± 0.04	van Breukelen et al. (2023) ^c^
Weekly mean methane concentration, ppm	0.32 ± 0.02	van Breukelen et al. (2023) ^c^

^1^Using Blaxter and Clapperton (1965) equations.

^2^Using Johnson et al. (1995) equations.

^3^Using International Panel on Climate Change (2006) equations.

^4^Expected methane production obtained by the regression of MPR on DMI.

^5^Using Kennedy et al. (1993) equations for the residual phenotypic regressions of a trivariate analysis of MPR, DMI, and weight.

^6^Using Kennedy et al. (1993) equations for the residual genetic regressions of a trivariate analysis of MPR, DMI, and weight.

^7^Using Dijkstra et al. (2011) equations.

^8^Using Dijkstra et al. (2011) equations excluding fatty acids with a difference >40% between data sets.

^9^Using Dijkstra et al. (2011) equations excluding fatty acids with a difference >40% between data sets and with concentrations <1 g/100 g fat.

^10^Using de Haas et al. (2011) equations.

^11^Using milk mid-infrared spectrometry in first lactation and Vanlierde et al. (2015) equations.

^a^Angus cattle.

^b^Holstein dairy cattle.

^c^Dairy cows.

**Table 3.** Phenotypic and genetic correlations involving enteric methane emissions phenotypes

Trait 1	Trait 2	Correlation ± SE	Study	Type of correlation
Methane production rate (g/d)	Methane yield (g/ kg DMI)	0.68 ± 0.02	Donoghue et al. (2016)	Phenotypic
		0.50 ± 0.14	Donoghue et al. (2016)	Genetic
		0.72 ± 0.02	Herd et al. (2014)	Phenotypic
	CH4:CO_2_ ratio	0.83 ± 0.14	Lassen and Løvendahl (2016)	Genetic
	Fat- and protein-corrected milk	0.43 ± 0.10	Lassen and Løvendahl (2016)	Genetic
	Residual methane production	0.65 to 0.79^*^	Herd et al. (2014)	Phenotypic
	Dry matter intake	0.65 ± 0.02	Herd et al. (2014)	Phenotypic
	Birth weight	0.26 ± 0.04	Donoghue et al. (2016)	Phenotypic
		0.36 ± 0.18	Donoghue et al. (2016)	Genetic
		0.19 ± 0.05	Herd et al. (2014)	Phenotypic
	Weaning weight	0.53 ± 0.03	Donoghue et al. (2016)	Phenotypic
		0.84 ± 0.09	Donoghue et al. (2016)	Genetic
		0.50 ± 0.04	Herd et al. (2014)	Phenotypic
	Yearling weight	0.61 ± 0.03	Donoghue et al. (2016)	Phenotypic
		0.86 ± 0.06	Donoghue et al. (2016)	Genetic
		0.57 ± 0.03	Herd et al. (2014)	Phenotypic
	Final weight	0.56 ± 0.03	Donoghue et al. (2016)	Phenotypic
		0.79 ± 0.08	Donoghue et al. (2016)	Genetic
		0.49 ± 0.05	Herd et al. (2014)	Phenotypic
	Ribeye area	0.40 ± 0.16	Donoghue et al. (2016)	Genetic
		0.29 ± 0.04	Herd et al. (2014)	Phenotypic
Methane yield (g/ kg DMI)	Dry matter intake	−0.04 ± 0.18	Donoghue et al. (2016)	Genetic
		−0.02 ± 0.04	Herd et al. (2014)	Phenotypic
	Residual methane production	0.82 to 0.97	Herd et al. (2014)	Phenotypic
	Birth weight	−0.03 ± 0.05	Herd et al. (2014)	Phenotypic
	Weaning weight	0.06 ± 0.05	Herd et al. (2014)	Phenotypic
	Yearling weight	0.11 ± 0.05	Herd et al. (2014)	Phenotypic
	Final weight	0.07 ± 0.06	Herd et al. (2014)	Phenotypic
	Ribeye area	0.01 ± 0.04	Herd et al. (2014)	Phenotypic
Predicted methane emissions	Residual feed intake	0.72	de Haas et al. (2011)	Phenotypic
	Dry matter intake	0.99	de Haas et al. (2011)	Phenotypic
	Fat- and protein-corrected milk	0.26	de Haas et al. (2011)	Phenotypic
CH4:CO_2_ ratio	Fat- and protein-corrected milk	0.37 ± 0.07	Lassen and Løvendahl (2016)	Genetic

^*^Dependent upon how RMP was calculated.

The cow-calf phase of the beef production cycle includes the largest number of animals compared to other phases within the beef value chain. The cow–calf phase is estimated to contribute 68% to 80% of the total greenhouse gas emissions from the beef life cycle (Beauchemin et al., 2010; Stackhouse-Lawson et al., 2012). Therefore, it is imperative that CH_4_ production heritability estimates are representative of the phase of the beef industry that produces the majority of CH_4_ emissions.

Hayes et al. (2016) derived genomic estimated breeding values (GEBV) for CH_4_ traits from a reference set of 747 Angus cattle, with a validation set on 273 additional Angus cattle. All animals in this study were born and raised on pasture, except for the period of CH_4_ measurement when they were fed a roughage diet consisting of alfalfa and oaten hay chaff in the respiration chamber. Methane production rate, MY, and four RMP traits were measured in respiration chambers. The estimated genomic heritability derived from only genomic information for MPR was 0.28 ± 0.06 and 0.20 ± 0.05 for MY (Hayes et al., 2016). The authors reported moderate accuracies of GEBV calculated from genomic best linear unbiased prediction for MPR and MY (0.32 ± 0.04 and 0.37 ± 0.09, respectively).

Manzanilla-Pech et al. (2016) estimated heritabilities for a variety of CH_4_ traits, measured with respiration chambers, for 1,020 Angus beef cattle (partially the same animals as Hayes et al., 2016). In this study, two validation populations of Holstein dairy cows collected with the SF_6_ tracer technique were also evaluated. The CH_4_ traits evaluated for the Angus population were MPR, MY, MI, residual phenotypic methane (RPM), and residual genetic methane (RGM). Residual phenotypic methane and RGM were calculated based on the residual phenotypic and genetic regressions of a trivariate analysis of MPR, DMI, and weight. The estimated heritabilities for MPR, MY, MI, RPM, and RGM in the Angus population were 0.30 ± 0.06, 0.20 ± 0.05, 0.25 ± 0.06, 0.19 ± 0.05, and 0.15 ± 0.05, respectively (Manzanilla-Pech et al., 2016). Heritabilities for the Holstein population were only evaluated for three CH_4_ traits and different values were observed. The estimated heritabilities for MPR, MY, and MI were 0.23, 0.30, and 0.42, respectively (Manzanilla-Pech et al., 2016). It is unknown whether the difference in heritability estimates was due to genetics or the smaller population size and higher associated standard errors (~0.23). The authors concluded that CH_4_ production is a moderately heritable trait, and several factors need to be evaluated to determine which trait is the “best” measure of CH_4_ emissions.

Donoghue et al. (2016) found genetic and phenotypic variance and covariance estimates for CH_4_ emission traits. Using largely the same animals as Manzanilla-Pech et al. (2016) and Hayes et al. (2016), this study included data from 1,046 Angus animals that were born and raised on pasture. Methane emissions were measured in a respiration chamber for 2 d while animals ate a roughage-based diet. The traits evaluated were MPR, MY, and four residual CH_4_ production traits as well as production traits such as birth weight (BW), weaning weight (WW), yearling weight (YW), and final weight (FW). Carcass traits such as ultrasound measures of longissimus muscle area (REA), rump fat depth, rib fat depth, and intramuscular fat were also included. One objective of this study was to estimate phenotypic and genetic correlations between CH_4_ and production traits (Donoghue et al., 2016). Donoghue et al. (2016) estimated the heritability of MPR and MY to be 0.27 ± 0.07 and 0.22 ± 0.06, respectively. All four forms of RMP had an estimated heritability of 0.19. Methane production rate and MY had a phenotypic correlation of 0.68 ± 0.02; this suggests that animals with high MPR also have high MY. The authors hypothesized that reducing MY will not impact DMI because the two traits are not genetically correlated (−0.04 ± 0.18); however, reducing MY will have an associative effect on MPR because the two traits have a moderate genetic correlation (0.50 ± 0.14). Interestingly, Donoghue et al. (2016) reported that MPR had a weaker phenotypic correlation with BW (0.26 ± 0.04) than later in life growth traits such as WW (0.53 ± 0.03), YW (0.61 ± 0.03), and FW (0.56 ± 0.03). Genetic correlations between MPR and production traits were moderate to strong: BW (0.36 ± 0.18), REA (0.40 ± 0.16), WW (0.84 ± 0.09), YW (0.86 ± 0.06), and FW (0.79 ± 0.08; Donoghue et al., 2016). The authors speculated that the moderate to strong genetic correlations between MPR and animal weight traits are likely due to the association between MPR and DMI. This means that reducing MPR will lead to a correlated reduction in animal weight for the progeny. Instead, the authors proposed the mitigation strategy of selecting reduced MY or residual CH_4_ because it could reduce CH_4_ production without a negative effect on DMI.

Although dairy cattle are different from beef cattle in many ways, heritability estimates from dairy cattle can give insight into beef cattle because they are both ruminant members of the Bovidae family. Three CH_4_ phenotypes including CH_4_:CO_2_ ratio, estimated CH_4_ production (g/d) over a week, and CH_4_ intensity (g CH_4_/L milk produced), were calculated for 3,121 Holstein dairy cows using an automatic milking system and FTIR detection (Lassen and Løvendahl, 2016). Both CH_4_ production and CH_4_ intensity had heritabilities of 0.21 ± 0.06 and CH_4_:CO_2_ ratio had a heritability of 0.16 ± 0.04 (Lassen and Løvendahl, 2016). Methane production and CH_4_:CO_2_ ratio had moderate genetic correlations to fat- and protein-corrected milk yield, (0.43 ± 0.10 and 0.37 ± 0.07, respectively; Lassen and Løvendahl, 2016). This suggests that CH_4_ production in dairy cattle is a heritable trait and that a strong genetic potential for milk production could yield greater CH_4_ emissions.

A large study of commercial dairy cows in the Netherlands estimated the heritability of both daily and weekly CH_4_ production (g/d) using measurements from a GreenFeed and CH_4_ concentration (ppm) from the SF6 tracer technique (van Breukelen et al., 2023). The heritability of daily and weekly CH_4_ production from GreenFeed measurements were 0.19 ± 0.02 and 0.33 ± 0.04, respectively (van Breukelen et al., 2023). The heritability of daily and weekly CH_4_ concentration from the SF6 tracer technique measurements were 0.18 ± 0.01 and 0.32 ± 0.02, respectively. The genetic correlation between CH_4_ production (g/d) measurements from a GreenFeed and CH_4_ concentration (ppm) from the SF_6_ tracer technique within the same time period was estimated (van Breukelen et al., 2023). The genetic correlation between daily CH_4_ production measured by a GreenFeed and daily CH_4_ concentration measured by the SF_6_ tracer technique was 0.71 ± 0.13. A similar correlation of 0.76 ± 0.13 was reported between the mean weekly CH_4_ production from a GreenFeed and mean weekly CH_4_ concentration from the SF_6_ tracer technique (van Breukelen et al., 2023). These high genetic correlations suggest that selection based on lower CH_4_ concentration measured by the SF_6_ tracer technique would result in lower CH_4_ production measured by the GreenFeed, on average. The authors suggested that measurements from both techniques could be used in a genetic evaluation together and data sharing of measurements from both techniques internationally would facilitate the creation of a larger genomic reference population.

van Engelen et al. (2015) used milk composition information (milk fatty acid profile) in three different MY prediction equations to estimate the MY of 1,905 Holstein-Friesian cows. The heritability estimates from the three different equations for CH_4_ yield were 0.12 ± 0.06, 0.20 ± 0.07, and 0.44 ± 0.10 (van Engelen et al., 2015). Methane yield based on milk fat composition is heritable.

Pickering et al. (2015) used feed intake, milk yield, live weight, and condition scores to predict CH_4_ emissions of 1,726 dairy cows. Predicted methane emissions (PME) were calculated daily from morning and evening milkings and then averaged for each week of lactation. Predicted methane emissions (g/d) had a mean heritability of 0.13 ± 0.04 across 44 wk of lactation (Pickering et al., 2015). The heritability of PME stayed relatively stable across the 44 wk of lactation measured. In this study, a laser CH_4_ detector was also used to collect repeated measurements from 57 cows. The repeatability of emissions from the laser CH_4_ detector within a lactation was 0.07 ± 0.08 and across lactations was 0.03 ± 0.08. The authors speculated that the low repeatability associated with the laser CH_4_ detector was due to the small sample size. Therefore, the laser CH_4_ detector was found to not be suitable for genetic prediction due to low repeatability and difficulty in obtaining a sufficient sample size (Pickering et al., 2015).

Methane emissions were predicted from feed intake, milk, and body weight data on 548 Holstein-Friesian heifers (de Haas et al., 2011). Predicted CH_4_ emissions gradually increased throughout lactation until emissions reached a plateau of approximately 400 g/d from mid-lactation until the end of lactation (de Haas et al., 2011). Predicted CH_4_ emissions had a heritability of 0.35 ± 0.12 for week 0 through week 42 of lactation and heritabilities estimates varied between the weeks of lactation from 0.29 to 0.42 with standard errors ranging from 0.10 to 0.12 (de Haas et al., 2011). Feed intake data collected from automated feeders were used to calculate RFI and DMI. Predicted CH_4_ emissions had a strong positive phenotypic correlation with RFI, suggesting that animals with lower RFI also would have lower PME (de Haas et al., 2011).

Kandel et al. (2017) studied two milk mid-infrared-based CH_4_ proxies: PME and log-transformed CH_4_ intensity (LMI). The fatty acid profile was predicted using mid-infrared spectrometry, and then an equation developed by Vanlierde et al. (2015) was used to find PME given the mid-infrared milk information (Kandel et al., 2017). Log-transformed CH_4_ intensity was calculated by log-transforming the ratio of PME over daily MY. Kandel et al. (2017) studied both first (*n* = 56,957) and second (*n* = 34,992) parity cows. The heritability of PME was moderate and slightly decreased from first to second lactation, 0.25 ± 0.01 and 0.22 ± 0.01, respectively (Kandel et al., 2017). The heritability of LMI was 0.18 ± 0.01 for first lactation and 0.17 ± 0.02 for second lactation (Kandel et al., 2017). Between the first and second lactation, PME increased (433 g/d vs. 453 g/d) while LMI decreased (2.93 vs. 2.86; Kandel et al., 2017). The authors suggested that the rankings of animals were similar between the two lactations based on the high Spearman correlation values for PME and LMI, 0.92 and 0.95, respectively. Kandel et al. (2017) explained that the differences in values observed between first and second lactation were due to changes in feed intake, feed efficiency, energy partitioning, and milk production. Although PME is lowly heritable, it is a problematic trait to use for genetic selection. Predicted CH_4_ is calculated using various component traits, therefore, those component traits change with selection rather than by directly selecting for CH_4_.

Although a different species, sheep are grazing ruminant animals that also produce CH_4_. Sheep are typically less expensive to manage and are easier to handle, offering a potential proxy for cattle in CH_4_ emissions research. Robinson et al. (2010) evaluated 708 grazing ewes for 1-h CH_4_ emissions using a sealed polycarbonate booth. The heritability of 1-h CH_4_ production (dL/h) after adjustments for live weight was 0.13 with a repeatability of 0.32 (Robinson et al., 2010).

Pinares-Patiño et al. (2013) measured MPR and MY from 1225 sheep in respiration chambers. The heritability of MPR and MY was 0.29 ± 0.05 and 0.13 ± 0.03, respectively (Pinares-Patiño et al., 2013). Measurements in respiration chambers were repeated 14 d later to assess repeatability. Methane production and MY had repeatabilities of 0.55 ± 0.02 and 0.26 ± 0.02, respectively (Pinares-Patiño et al., 2013). The results of this study suggest that CH_4_ emission traits are heritable and repeatable for sheep.

### Selection Strategies

The objective is to reduce CH_4_ emissions from beef cattle to optimize productivity and profitability with sustainability. However, CH_4_ production is a natural digestive process of ruminants that allows cattle to digest and ferment plant material. Therefore, it is vital that the optimum balance between CH_4_ production and animal productivity is reached, as maximum productivity and minimum emissions are likely incompatible.

High feed intake is associated with high MPR in ruminants (Blaxter and Clapperton, 1965). Production traits such as growth are highly correlated with feed intake (Arthur et al., 2001). Therefore, reducing MPR could have an unfavorable impact on animal productivity due to the correlation with feed intake. Herd et al. (2014) evaluated several ways to measure CH_4_ including MPR, MY, and four forms of RMP, and estimated the phenotypic relationships between the CH_4_ traits and the production traits. Methane production rate was positively correlated with DMI, MY, RMP, growth traits, and body composition traits (0.65 ± 0.02; 0.72 ± 0.02; 0.65 to 0.79; 0.19 to 0.57; 0.13 to 0.29). However, MY was not correlated with DMI, growth traits, or body composition traits (−0.02 ± 0.04; −0.03 to 0.11; 0.01 to 0.06). All four forms of RMP were strongly correlated with MY (0.82 to 0.97; Herd et al., 2014). These results suggest that reducing MPR as a mitigation strategy would have a negative impact on growth and body composition traits. However, MY was not correlated with DMI but was positively correlated with MPR. This suggests that reducing MY would have no effect on DMI or animal productivity but would have a correlated reducing effect on MPR. However, ratio traits are undesirable in genetic evaluations because the changes in component traits are difficult to predict for future generations (Arthur et al., 2001). Additionally, using a ratio trait in genetic evaluation has the potential to place greater than expected emphasis on the trait with higher genetic variance (Gunsett, 1984).

Instead of MY, selection for one of the RMP component traits has been proposed. An RMP trait independent from DMI may be the best trait to incorporate into selection strategies (Herd et al., 2014). However, residual traits used for genetic evaluation have disadvantages. Using initial variables to calculate other variables, as done for RMP, can reduce variation leading to ineffective animal ranking which is critical for genetic evaluation (van der Werf, 2004). There are tradeoffs for both residual and ratio traits and the correlations reported by Herd et al. (2014) are phenotypic correlations. Therefore, future research on the genetic relationships between these traits and growth traits is needed to decide which CH_4_ trait should be incorporated into selection strategies.

Development of a selection index for CH_4_ production would be the most advantageous mitigation strategy. A well-constructed index with properly weighted traits would allow for optimum selection to reduce CH_4_ production without compromising important production traits, such as DMI. One of the biggest difficulties of constructing a selection index is assigning the appropriate economic weighting to each trait in the index. Currently, the economic value of enteric CH_4_ emissions is unknown and the price of carbon is not globally realized (Lakamp et al., 2022). Further research is required in this area to define economic values for CH_4_ production and evaluate its weighting in a selection index. Additionally, continued CH_4_ phenotype data collection is needed for large-scale genetic evaluations to establish genetic correlations between CH_4_ production and other economically important traits (LaKamp et al., 2022). This would allow for the construction of a properly weighted selection index to reduce CH_4_ without economic losses from reduced performance.

## Conclusion

Methane is a greenhouse gas with adverse effects on the environment due to the warming potential of the atmosphere. Enteric fermentation from ruminant animals is a source of CH_4_ production and represents an energetic loss for that animal. A wide variety of dietary factors as well as genetics influence the amount of CH_4_ produced from cattle. Several methods exist to quantify CH_4_ emissions from cattle, including respiration calorimetry, the sulfur-hexafluoride tracer technique, prediction models, and GreenFeed. The accuracy of each technique and comparisons between the techniques have shown that generally, respiration chambers and the GreenFeed report similar CH_4_ emissions. In general, the SF6 tracer technique estimates emissions higher than those from respiration chambers and the GreenFeed. Prediction models have distinct disadvantages but can be a useful tool to estimate CH_4_ emissions from large groups of animals with accurate feed intake information, rather than for individual animals as required for genetic evaluation. Methane emissions are a heritable trait which allows genetic progress to be made with genetic selection. Although CH_4_ production represents an energetic loss to animals, it is also a component of fermentation- an important digestive process. Therefore, adverse effects on production should be considered when employing selection to reduce CH_4_ production. There are moderate genetic correlations between MPR and performance traits; however, selection for animals with desirable breeding values for performance but lower emissions should be possible and would likely be most efficacious using a well-constructed selection index.
